# Identification, Verification and Pathway Enrichment Analysis of Prognosis-Related Immune Genes in Patients With Hepatocellular Carcinoma

**DOI:** 10.3389/fonc.2021.695001

**Published:** 2021-09-20

**Authors:** Zhipeng Zhu, Mengyu Song, Wenhao Li, Mengying Li, Sihan Chen, Bo Chen

**Affiliations:** ^1^Department of General Surgery, The First Affiliated Hospital of Anhui Medical University, Hefei, China; ^2^Department of Clinical Medicine, The First Clinical College, Anhui Medical University, Hefei, China

**Keywords:** immune genes, bioinformatics analysis, prognostic, signature, hepatocellular carcinoma

## Abstract

Hepatocellular carcinoma is a common malignant tumor with poor prognosis, poor treatment effect, and lack of effective biomarkers. In this study, bioinformatics analysis of immune-related genes of hepatocellular carcinoma was used to construct a multi-gene combined marker that can predict the prognosis of patients. The RNA expression data of hepatocellular carcinoma were downloaded from The Cancer Genome Atlas (TCGA) database, and immune-related genes were obtained from the IMMPORT database. Differential analysis was performed by Wilcox test to obtain differentially expressed genes. Univariate Cox regression analysis, lasso regression analysis and multivariate Cox regression analysis were performed to establish a prognostic model of immune genes, a total of 5 genes (*HDAC1, BIRC5, SPP1, STC2, NR6A1*) were identified to construct the models. The expression levels of 5 genes in HCC tissues were significantly different from those in paracancerous tissues. The Kaplan-Meier survival curve showed that the risk score calculated according to the prognostic model was significantly related to the overall survival (OS) of HCC. The receiver operating characteristic (ROC) curve confirmed that the prognostic model had high accuracy. Independent prognostic analysis was performed to prove that the risk value can be used as an independent prognostic factor. Then, the gene expression data of hepatocellular carcinoma in the ICGC database was used as a validation data set for the verification of the above steps. In addition, we used the CIBERSORT software and TIMER database to conduct immune infiltration research, and the results showed that the five genes of the model and the risk score have a certain correlation with the content of immune cells. Moreover, through Gene Set Enrichment Analysis (GSEA) and the construction of protein interaction networks, we found that the p53-mediated signal transduction pathway is a potentially important signal pathway for hepatocellular carcinoma and is positively regulated by certain genes in the prognostic model. In conclusion, this study provides potential targets for predicting the prognosis and treatment of hepatocellular carcinoma patients, and also provides new ideas about the correlation between immune genes and potential pathways of hepatocellular carcinoma.

## Introduction

Liver cancer is the most common cause of cancer death worldwide and is the only one of the top five deadliest cancers to have an annual percentage increase in occurrence. The incidence of liver cancer is rising faster than any other cancer, and pancreatic and liver cancer have the lowest survival rates compared with other cancers (1). Hepatocellular carcinoma (HCC), the most common liver cancer, accounts for 80% of all liver cancer cases (2), is the third leading cause of cancer-related mortality worldwide (3). Because of the high heterogeneity of hepatocellular carcinoma, there is an urgent need for biomarkers that can predict the prognosis of patients. Alpha-fetoprotein (AFP), Neutrophil-to-Lymphocyte Ratio (NLR), Glypican-3 (GPC3) and other indicators were considered to indicate the prognosis of patients with hepatocellular carcinoma, but because of the complex molecular mechanism and strong heterogeneity of hepatocellular carcinoma, these indicators still have some limitations (4). However, due to the complex molecular mechanism and high heterogeneity of hepatocellular carcinoma, the predictive ability of traditional prognostic indicators is slightly inadequate. Therefore, it is necessary to find suitable biomarkers to effectively evaluate the prognosis of patients with HCC.

Recent studies have shown that immunity is closely related to the occurrence and development of tumors, and the immune system has been proved to be a decisive factor in the occurrence and development of cancer (5). The molecular mechanism of the interaction between tumor and immune system provides a new way for the treatment of tumor (6, 7). Immunotherapy for cancer can specifically fight against malignant cells, and has become one of the most promising cancer therapies (8). Inhibitory therapy for PD-1/PD-L1 immune checkpoint has been used to treat hepatocellular carcinoma (9), and the therapeutic effect is good. These studies can reflect that immune-related molecules may play an important role in tumor therapy and have the potential to become therapeutic targets. The studies of Zhang et al. have shown that the molecular map of immune components in tumor microenvironment is of great value as a biomarker of prognosis (10). In recent years, due to the maturity of high-throughput sequencing technology, a series of changes in tumor tissue genome and normal tissue genome have been excavated. Several studies in different types of cancer, including ovarian cancer, cervical cancer and lung squamous cell cancer (11–13), immune-related genes were used to establish tumor prognostic models, which further reflects the potential of immune-related genes to become tumor prognostic markers.

In this study, we used the immune genes provided by IMMPORT database to obtain the data of differentially expressed immune genes in the TCGA-LIHC cohort. Through univariate cox regression analysis, lasso regression analysis and multivariate cox regression analysis, the immune gene risk score model related to prognosis was established, including *HDAC1*, *BIRC5*, *SPP1*, *STC2* and *NR6A1* five genes. According to the median risk score, patients were divided into high risk group and low risk group, and then Kaplan-Meier survival curve and ROC curve were constructed to evaluate the predictive value of risk score for the prognosis of patients with hepatocellular carcinoma. We verified the predictive ability of the prognostic model in the ICGC liver cancer cohort, and explored the expression patterns and immune infiltration levels of the five genes in the model. Finally, the relationship between prognosis-related immune genes and the potential pathway of hepatocellular carcinoma was explored by bioenrichment analysis.

## Materials and Methods

### Data Acquisition

The RNA-seq data and clinical data of the hepatocellular carcinoma dataset were downloaded from the TCGA (https://portal.gdc.cancer.gov/) database and the ICGC database (https://dcc.icgc.org/).We downloaded the gene list of the IMMPORT database (https://www.immport.org/shared/home/) to obtain immune-related genes. The genes related to the signal transduction pathway by p53 class mediator were obtained by gene set enrichment analysis (GSEA) of RNA-seq data from hepatocellular carcinoma data set.We obtained the immune cell content file of the TCGA sample from the Timer database (https://cistrome.shinyapps.io/timer/). The software CIBERSORT was used to estimate the composition of immune cells in hepatocellular carcinoma tissues.We used R (4. 0.2) to standardize data and analyzed differential expression to obtain significantly different immune genes and genes related to the signal transduction pathway of p53 mediators. The Cytoscape software was used to construct the PPI network.

### Construction of the Prognostic Models

After standardizing the data downloaded from TCGA database and ICGC database, the expression data was obtained. The wilcox test was used to analyze the difference of gene expression data obtained from TCGA database, and then significant differential genes (DEGs) were screened (screening conditions: | logFC | > 1, FDR (False Discovery Rate) <0.05). Immune-related genes were obtained from the gene list in the IMMPORT database, and the differential immune genes were obtained by intersection with the DEGs. The differential immune genes were intersected with the genes in ICGC hepatocellular carcinoma data, and the expression data of hepatocellular carcinoma differential immune genes in TCGA and ICGC databases were obtained. Univariate COX risk regression analysis was used to screen immune genes that are significantly related to overall survival (OS) in the TCGA hepatocellular carcinoma data set. Lasso regression analysis was used to eliminate the highly correlated genes among these prognostic genes to avoid the problem of overfitting, the cross-validation error of the remaining genes is minimal. The “survival” R package was used to perform multivariate COX risk regression analysis, and the constructed prognosis model of hepatocellular carcinoma immune genes was based on data from the TCGA database. The patient’s risk score was calculated by the prognostic model: risk score=∑XJ*coefJ, “XJ” is the relative expression level of each immune gene in the model; “coefJ” is the correlation coefficient of the gene. Patients in the two databases were divided into two groups (high-risk group and low-risk group) with their respective median risk score as the critical value.

### Evaluation of the Accuracy of the Prognostic Model and Verification of External Databases

The risk score of hepatocellular carcinoma patients in the TCGA database and the gene expression data in the model are combined to output a risk file. In order to observe the accuracy of the model and judge the predictive ability of risk score to the prognosis of patients with hepatocellular carcinoma. In order to observe whether the risk score can effectively predict the clinical prognosis of patients with hepatocellular carcinoma, Kaplan-Meier (K-M) survival curve was established to analyze the difference in survival between patients in the high-risk group and the low-risk group. The accuracy of the prognostic model was evaluated by constructing the ROC curve. In order to explore whether the risk score of patients with hepatocellular carcinoma based on the prognostic model can be used as an independent prognostic factor, age and other clinical traits and the risk score were used for univariate independent prognostic analysis and multivariate independent prognostic analysis. The gene expression data from the ICGC database were substituted into the model as a verification set, and the above process was repeated to verify the prognostic model.

### Verification of the Prognostic Genes Expression and Immune Infiltration

The gene expression was verified by extracting the expression of five prognostic genes in TCGA database and ICGC database. The immunohistochemical results of prognostic genes were obtained by searching the HPA database. The content of immune cells in the sample was obtained by using TCGA hepatocellular carcinoma expression data, and the correlation between prognostic genes and immune infiltration was obtained. The content of immune cells was obtained from the TIMER database, and the correlation between the risk value and the content of immune cells was further obtained.

### GSEA of the Hepatocellular Carcinoma Data Set and Construction of PPI Network

We used GSEA software to perform enrichment analysis of hepatocellular carcinoma dataset to obtain genes related to the signal transduction pathway of p53 mediators, and screened these genes to obtain differentially expressed genes. The resulting genes were used for correlation analysis of prognostic-related immune genes, and the PPI network was constructed according to the results of the analysis.

## Results

### Differential Gene Expression Analysis

**Figure 1:**
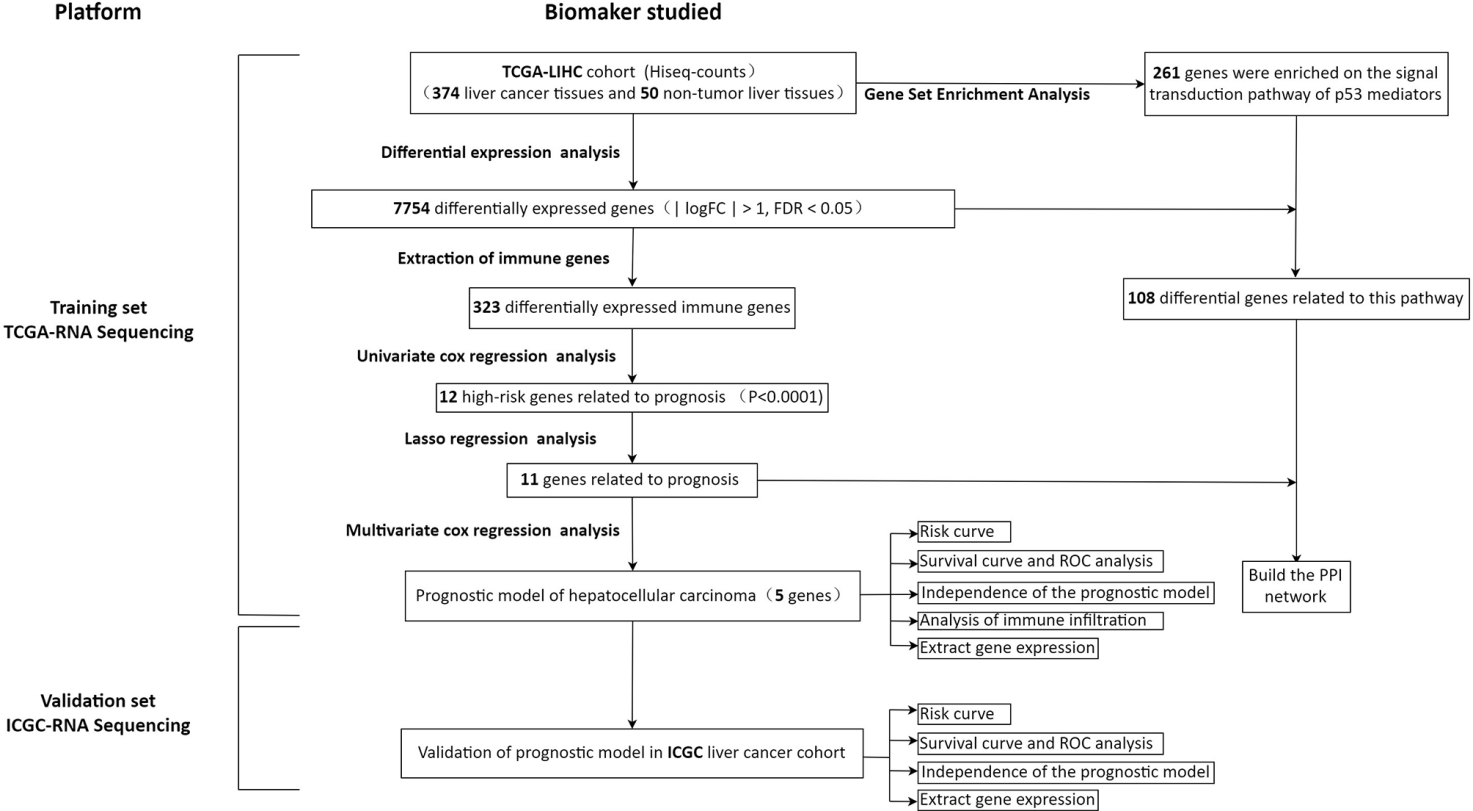
Flow chart of this study.

**Figure 2:**
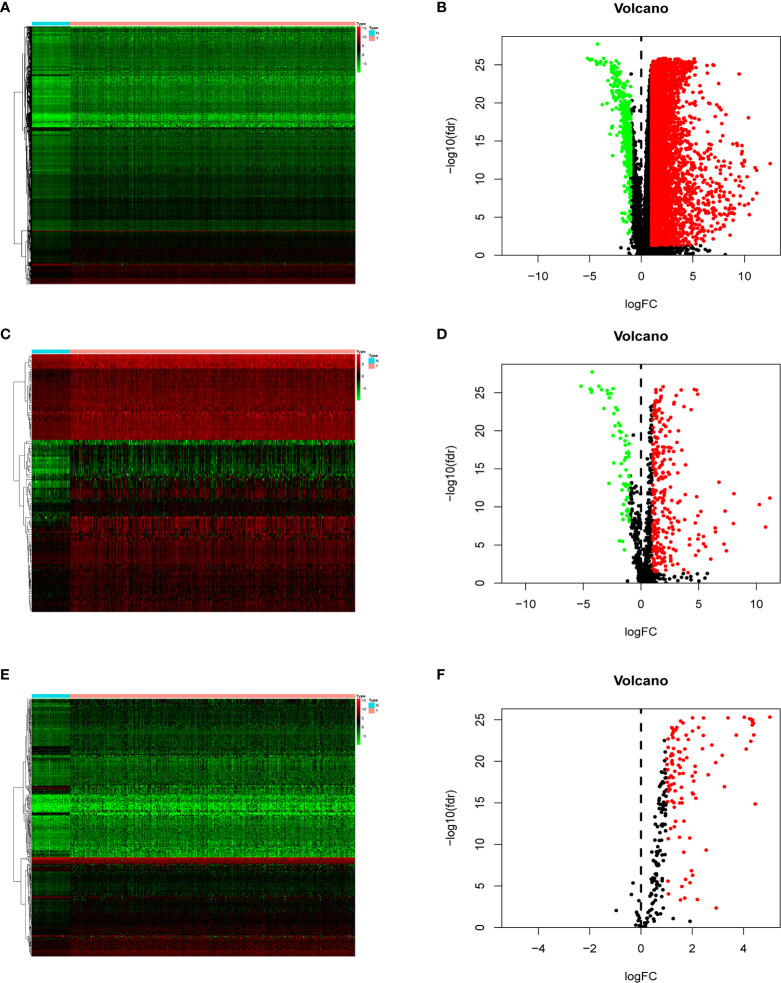
Heat map **(A)** and volcano map **(B)** of differentially expressed genes; heat map **(C)** and volcano map **(D)** of immune differential genes; heat map **(E)** and volcano map **(F)** of differentially expressed genes related to the signal transduction pathway of P53 mediators. The abscissa of the heat map represents the sample: the blue area represents the adjacent tissue, the red area represents the hepatocellular carcinoma tissue; the ordinate represents the gene. The red dots on the volcano map represent genes whose expression levels are up-regulated, and the green dots represent genes whose expression levels are down-regulated.

In order to show our research process more clearly, an analysis flow chart is used to describe (**Figure 1**). The research team obtained the mRNA expression profile and clinical information of patients with hepatocellular carcinoma from the TCGA database. The TCGA-LIHC cohort included 374 hepatocellular carcinoma tissues and 50 non-tumor liver tissues. Wilcoxon test was used to analyze the differences of all RNA sequencing data (screening condition: | logFC | > 1, FDR < 0.05), and 7754 differentially expressed genes (DEGs) were obtained, and used the “pheatmap” package in R to draw a heat map and a volcano map (**Figures 2A, B**). These DEGs were intersected with the immune genes obtained from the IMMPORT database, and 333 differentially expressed immune genes were obtained, and also used the “pheatmap” package to draw a heat map and a volcano map (**Figures 2C, D**). The 333 genes based on the TCGA database were intersected with the gene expression data of hepatocellular carcinoma in the ICGA database, and 323 differentially expressed immune genes were obtained.

### The Accurate Prognostic Gene Model of Hepatocellular Carcinoma Was Constructed

**Figure 3:**
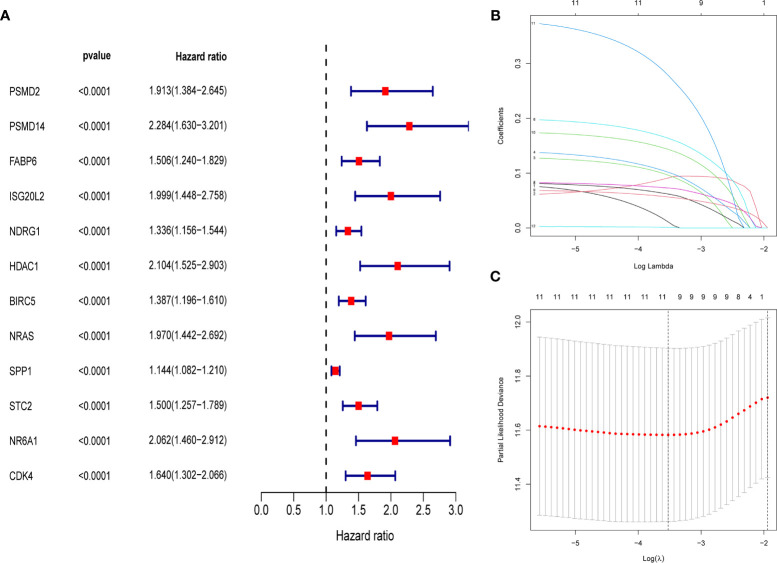
**(A)** Forest map of 12 immune genes related to the prognosis of hepatocellular carcinoma, analyzed by univariate Cox regression, all 12 genes are high risk genes (HR >1, *P* < 0.0001). **(B)** LASSO coefficient spectrum of 12 immune genes, Generate a coefficient distribution map for a logarithmic (λ) sequence. **(C)** Selecting the best parameters for LIHC in the LASSO model (λ).

First, merge the expression data of 323 genes with survival information, which includes survival time and survival status; After that, univariate cox regression analysis was performed on 323 differential immune genes, and 12 high-risk genes(Hazard ratio > 1) related to prognosis were obtained (**Figure 3A**). High-risk genes mean that the higher the expression level of the gene, the greater the risk of the patient and the shorter the survival time. These 12 prognostic genes all satisfy *P* value <0.0001. In order to prevent over-fitting when constructing a prognostic model of hepatocellular carcinoma, the 12 prognostic-related genes were subjected to the lasso regression analysis, and the genes with high correlation were deleted. 11 genes (**Supplementary Data Sheet 1**) were obtained when the cross-validation error was the smallest (**Figures 3B, C**). After that, the “survival” package was used to perform multivariate cox regression analysis on these 11 genes in R, and finally 5 genes(*HDAC1*ˎ*BIRC5*ˎ*SPP1*ˎ*STC2*ˎ*NR6A1*) related to the prognosis of LIHC were obtained to construct a prognostic model. Use the relative expression level of each gene in the prognostic model and the correlation coefficient of each gene to calculate the patient’s risk score, risk score = (0.293753798* expression level of *HDAC1*) + (0.140659127 * expression level of *BIRC5*) + (0.083311679 * expression level of *SPP1*) + (0.245656937 * expression level of *STC2*) + (0.404582055 * expression level of *NR6A1*). The correlation coefficient of each gene was shown in **Table 1**.

**Table 1:** Genes contained in the prognostic model of hepatocellular carcinoma.

Gene symbol	Full name	Coef	HR	*P* value
*HDAC1*	Histone Deacetylase 1	0.293754	1.341454	0.127148
*BIRC5*	Baculoviral IAP Repeat Containing 5	0.140659	1.151032	0.125742
*SPP1*	Secreted Phosphoprotein 1	0.083312	1.086881	0.011504
*STC2*	Stanniocalcin 2	0.245657	1.278461	0.020805
*NR6A1*	Nuclear Receptor Subfamily 6 Group A Member 1	0.404582	1.498676	0.039342

**Figure 4:**
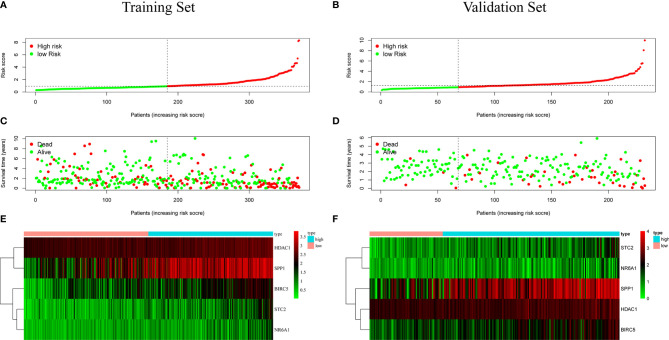
**(A, B)** Distribution of risk score in patients with hepatocellular carcinoma. The black dotted line serves as the dividing line between the high-risk group and the low-risk group. **(C, D)** Diagram of the relationship between risk score and patient survival time. **(E, F)** Heat map of five immune genes in prognostic model, the abscissa represents the sample: the red area is the low-risk group, and the blue area is the high-risk group. The result of **(A, C, E)** is based on TCGA data(training set), and the result of **(B, D, F)** is based on ICGC data (validation set).

**Figure 5:**
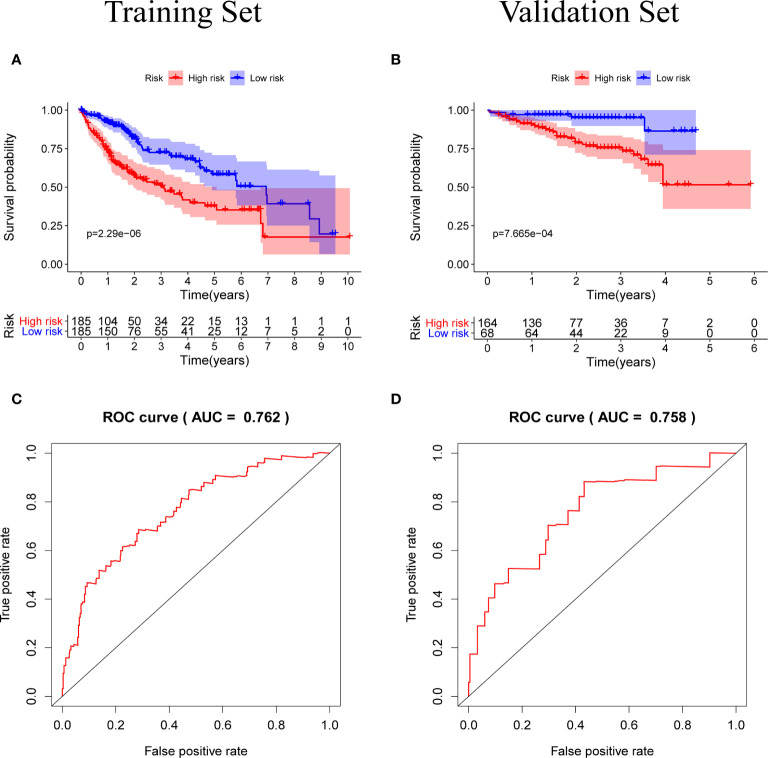
**(A, B)** Kaplan-Meier survival curves of the training set and the validation set, the survival prognosis of the patients in the high-risk group was significantly worse than that of the patients in the low-risk group (*P* < 0.05). **(C, D)** The ROC curve of the prognostic model, the results of the training set (AUC = 0.762) and the validation set (AUC = 0.758) show that the predictive ability of the model is good. (The meaning of AUC value: 0.5-0.7 indicates acceptable predictive ability, 0.7-0.9 indicates good predictive ability, >0.9 indicates excellent predictive ability).

Patients were classified according to the calculated median risk score. 185 patients in the TCGA database were divided into high risk group and 185 patients were divided into low risk group (**Supplementary Data Sheet 2**). **Figure 4A** shows the distribution of the risk scores of hepatocellular carcinoma patients from low to high. **Figure 4B** shows that with the increase of the risk score, the prognosis of the patient is worse. Draw a heat map of 5 genes in the model to show the expression profiles of patients in high-risk and low-risk groups (**Figure 4C**). The results showed that patients in the high-risk group were more likely to express these five genes. The Kaplan-Meier survival curve (**Figure 5A**) drawn with “survival” package and “survminer” package in R showed a significant difference in survival prognosis between the two groups (*P*=2. 29e-06): the five-year survival rate was 37. 8% (95%CI:28. 40%~50.2%) in the high-risk group and 58. 2% (95%CI:47. 94% ~ 70.8%) in the low-risk group. The predictive ability of the model used the “survivalROC” package to draw the ROC curve and calculate the AUC value for evaluation. The results show that the AUC based on the risk score obtained by the model was 0.764, which shows that the prediction accuracy of the model is good. (**Figure 5B**). The patients in the TCGA-LIHC cohort were divided into training set and verification set according to the proportion of 7:3. The internal verification results indicated that the predictive ability of our risk score model was good, and the survival probability of patients in the high-risk group was significantly worse than that in the low-risk group (**Figure S1**).

### The Prognostic Model Is an Independent Prognostic Factor

**Figure 6:**
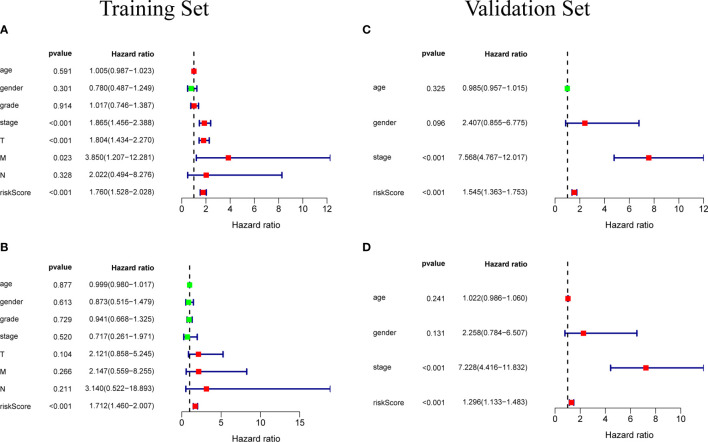
**(A)** Univariate independent prognostic analysis of the training set. Forest plot of the association between risk factors and overall survival of patients. **(B)** Multivariate independent prognostic analysis of the training set. The risk score based on the prognostic model can be used as an independent prognostic factor for hepatocellular carcinoma. **(C, D)** Independent prognostic analysis of univariate and multifactorial factors in the validation set. The forest map shows that the risk score can also be used as an independent prognostic factor for hepatocellular carcinoma in the validation set.

Univariate independent prognostic analysis and multivariate independent prognostic analysis were used to evaluate the independent predictive value of a prognostic model composed of five genes in 235 patients with complete clinical information from the TCGA-LIHC cohort. Univariate independent prognostic analysis showed that the risk score of TCGA-LIHC had a certain predictive value for prognosis, and was significantly correlated with overall survival (OS) (HR=1. 760, 95%CI: 1. 528~2. 028, *P*<0.001) (**Figure 6A**). Multivariate independent prognostic analysis showed that risk score was an independent prognostic factor related to OS (HR=1. 712, 95%CI: 1. 460~2. 007, *P*<0.001) (**Figure 6B**). The above results indicate that the risk score obtained through the prognostic model can be used as an independent prognostic factor in clinical practice and has important clinical significance.

### External Verification of Prognostic Model Using ICGC Database

In order to test the general applicability of the prognostic model, it is necessary to use data from different sources for external verification. Through the analysis of hepatocellular carcinoma data in the ICGC database, to verify the predictive ability of the prognostic model in the prognosis of hepatocellular carcinoma patients. According to the median value of the risk score calculated by the prognosis model, 232 patients in the ICGC database were divided, 164 patients were classified into the high-risk group, and 68 patients were classified into the low-risk group (**Supplementary Data Sheet 3**). The risk score distribution map (**Figure 4D**), survival status map (**Figure 4E**) and gene heat map (**Figure 4F**) drawn based on the hepatocellular carcinoma data of the ICGC database also showed that the higher the risk score, the worse the prognosis of the patient. Consistent with the TCGA results, the Kaplan-Meier survival curve (**Figure 5C**) showed that the survival prognosis of patients in the high-risk group and the low-risk group in the ICGA data was significantly different (*P*=7. 665e−04): the five-year survival rate of the high-risk group was 51. 6% (95%CI: 35. 9%~74. 1%), the five-year survival rate of the low-risk group was 86. 4% (95%CI: 71. 1%~100%). The ROC curve shows that the AUC of risk score based on the prognostic model is 0.785, which indicates that the prognostic model still has good predictive ability in the ICGC database (**Figure 5D**). The data of 232 patients with hepatocellular carcinoma with complete clinical information in the ICGC database were used for univariate independent prognostic analysis and multivariate independent prognostic analysis. The results showed that the risk score was still significantly correlated with OS and can be used as an independent prognostic factor (**Figures 6C, D**).

### The Correlation Between Prognostic Models and Immune Cells

**Figure 7:**
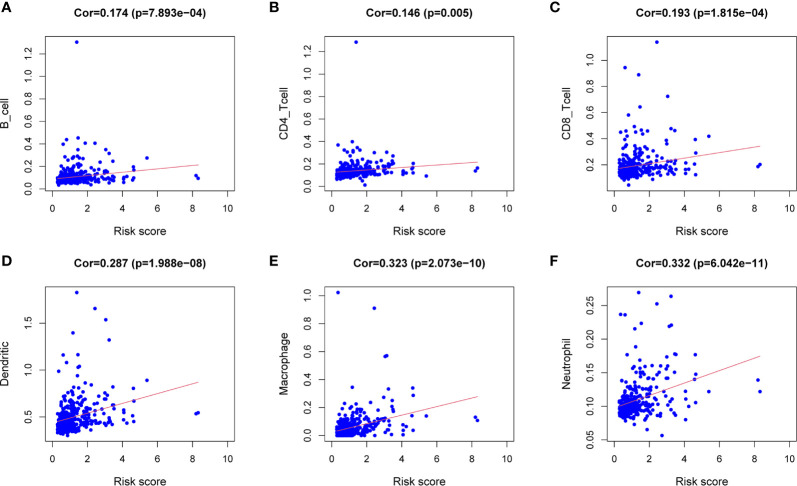
Correlation analysis between the expression of immune microenvironment cells and risk score. There are six types of immune cells involved in the correlation analysis, including B cells **(A),** CD4^+^ T cells **(B),** CD8^+^ T cells **(C),** dendritic cells **(D),** macrophages **(E),** and neutrophils **(F)**.

**Figure 8:**
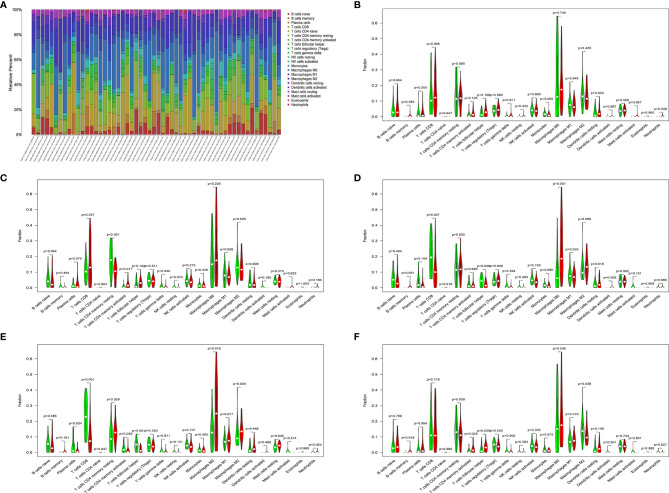
**(A)** Histogram of immune cells. The abscissa is the sample selected from the TCGA-LIHC cohort (screening condition: *P* < 0.05), and the vertical row represents the composition of various immune cells in the sample. Each color represents a different cell type. **(B–F)** Violin diagrams of five genes for constructing the model, including *HDAC1* **(B),** *BIRC5* **(C),** *SPP1* **(D),** *STC2* **(E)** and *NR6A1* **(F)**. There are 22 kinds of immune cells on the abscissa, and the ordinate represents the content of immune cells. The red area represents the high expression group of genes, and the green area represents the low expression group of genes.

Download the immune cell content files of TCGA database samples from the TIMER database, including six types of immune cells: B cells, CD4^+^ T cells, CD8^+^ T cells, neutrophils, macrophages, and dendritic cells. The correlation analysis between the content of the immune cells of the samples and the risk scores of the samples showed that the risk scores were positively correlated with the six immune cells (cor>0, *P*<0.05) (**Figures 7A–F**). In order to further study the relationship between the prognostic model composed of five genes and immune cells, the gene expression data of the samples in the TCGA-LIHC cohort and the gene expression profiles of 22 kinds of immune cells were analyzed by CIBERSORT software, and the contents of various immune cells in the samples were estimated (**Figure 8A**). According to the expression level of each gene, patients were divided into high expression group and low expression group. Combining the content of various immune cells in the sample, use the “limma” software package and the “vioplot” software package to perform differential analysis in R to determine whether different immune cells have significant differences in the high gene expression group and the low gene expression group. The results showed that there were significant differences in some immune cells between the high expression group and low expression group of certain genes (*P*<0.05) (**Figures 8B–F** and **Table 2**).

**Table 2:** Details of immune cells with different contents between the high gene expression group and the low gene expression group.

Gene	Immune Cell	*P* value
*BIRC5*	resting memory CD4^+^ T cells	0.000828038
	activated memory CD4^+^ T cells	0.01692837
*NR6A1*	activated memory CD4 T^+^ cells	0.028519812
	helper follicular T cells	0.029346974
	M2 Macrophages	0.038128193
*SPP1*	CD8^+^ T cells	0.007467591
	helper follicular T cells	0.028402223
	M0 Macrophages	0.000583091
*STC2*	Plasma cells	0.023723888
	CD8^+^ T cells	6. 88E-05
	resting memory CD4^+^ T cells	0.029168974
	activated memory CD4^+^ T cells	0.039169141
	helper follicular T cells	0.000338848
	M0 Macrophages	0.018507551
	M2 Macrophages	0.023511399
	resting Mast cells	0.044634306

### Validation of Five Gene Expression Patterns

**Figure 9:**
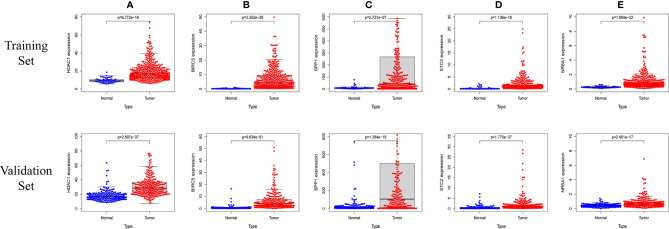
The five prognostic genes are upregulated in human HCC specimens: HDAC1 **(A)** , BIRC5 **(B)** , SPP1 **(C)** , STC2 **(D)** , NR6A1 **(E)**. Training Set: TCGA data showing the expression profiles of the five prognostic genes in normal liver (n = 50) *vs* tumor tissue (n = 371). Validation Set: ICGC data showing the expression profiles of the five prognostic genes in normal liver (n = 202) *vs* tumor tissue (n = 243).

**Figure 10:**
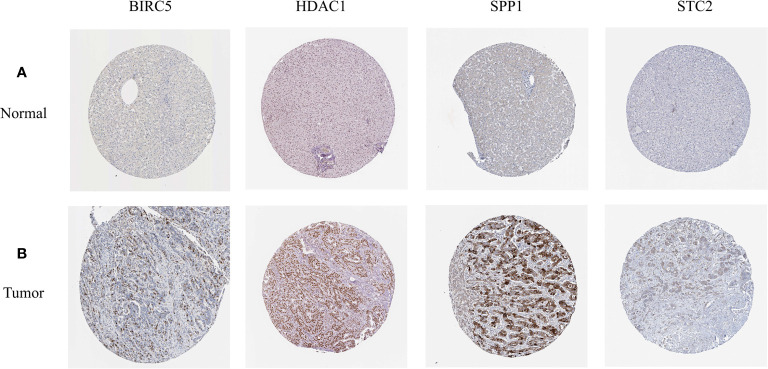
**(A, B)** The expression profiles of these five genes in normal liver tissues and hepatocellular carcinoma tissues. The results of immunohistochemistry were all from HPA database.

In order to verify the expression levels of the five genes that constitute the prognostic model of hepatocellular carcinoma, the “limma” and “beeswarm” packages were used in R to extract the expression levels of five genes from the gene expression matrix of the TCGA-LIHC cohort, and the results showed that the expression levels of the five genes in hepatocellular carcinoma tissues were significantly higher than those in adjacent tissues (**Figures 9A–E** Training Set). In order to further verify this result, we used the hepatocellular carcinoma data of the ICGC database to compare the expression levels of five genes again. Consistent with the results of TCGA, the expression levels of the five genes in hepatocellular carcinoma tissues (234 samples) were significantly higher than those in adjacent tissues (202 samples), and the results all met *P*<0.05 (**Figures 9A–E** Validation Set). In order to study the protein expression of the five genes in normal tissues and hepatocellular carcinoma tissues, we searched through the Human Protein Atlas (HPA) database and obtained the immunohistochemical results of the four genes (**Figures 10A, B**). The results of immunohistochemistry showed that the protein expression levels of *HDAC1*, *BIRC5*, *SPP1*, *STC2* in hepatocellular carcinoma tissues were higher than those in normal tissues. However, the immunohistochemical results of *NR6A1* were not found in the database.

### Gene Set Enrichment Analysis and Construction of PPI Network

**Figure 11:**
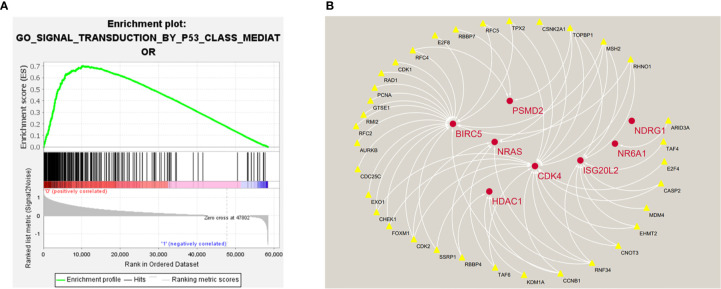
**(A)** Enrichment plot of 261 genes enriched on the signal transduction pathway of p53 class mediator. **(B)** Regulatory networks between prognosis-related immune genes and genes enriched in the pathway. There are three genes (*BIRC5*, *HDAC1*, *NR6A1*) in the model that participate in the hub of the network.

In order to explore the potential molecular pathways related to the prognostic markers of hepatocellular carcinoma, we used GSEA software to perform gene set enrichment analysis (GSEA) on 424 samples in the TCGA-LIHC cohort, including 374 hepatocellular carcinoma samples and 50 normal samples. The results of enrichment analysis showed that 261 genes were enriched on the signal transduction pathway of p53 mediators (**Figure 11A**). The enrichment results of this pathway showed |NES|>1, NOM *p*-val<0.05, FDR q-val<0.25, indicating that the gene set was meaningful. The results of enrichment analysis were reliable (NES=2. 0237732, FDRNES=6. 65 E-04), and the enrichment of genes was good (PNES=0.017). The 261 genes enriched in the signal transduction pathway of P53 mediators and the 7754 differentially expressed genes obtained by differential gene expression analysis were crossed, as a result, 108 genes were obtained. While these genes were enriched in the signal transduction pathway of P53 mediators, they were also differentially expressed in the TCGA-LIHC cohort. The heat maps and volcanoes of these genes are shown in **Figures 2E, F**. Twelve immune genes related to the prognosis of hepatocellular carcinoma obtained after univariate cox regression analysis. The 108 genes (**Supplementary Data Sheet 4**) and 12 immune genes were tested for correlation, cor=0.58 and pvalue=0.001 as the screening criteria to obtain the analysis results (**Table 3**). According to the analysis results, the Cytoscape software was used to draw the protein interaction network (**Figure 11B**).

**Table 3:** Details of the genes involved in the construction of regulatory networks.

P53 Gene	ImmuneGene	Cor	Pvalue	Regulation
*EHMT2*	*ISG20L2*	0.585473503	2. 03E-35	postive
	*CDK4*	0.593478392	1. 40E-36	postive
*RBBP7*	*PSMD2*	0.591027242	3. 20E-36	postive
*MSH2*	*ISG20L2*	0.671982407	6. 16E-50	postive
	*BIRC5*	0.604350044	3. 28E-38	postive
	*NRAS*	0.672605341	4. 65E-50	postive
	*CDK4*	0.631975097	1. 19E-42	postive
*RFC4*	*PSMD2*	0.589538975	5. 27E-36	postive
	*BIRC5*	0.763332173	7. 98E-72	postive
*CNOT3*	*ISG20L2*	0.614249869	9. 45E-40	postive
	*CDK4*	0.608447834	7. 67E-39	postive
*RAD1*	*BIRC5*	0.589519524	5. 31E-36	postive
*TAF6*	*NRAS*	0.591607607	2. 63E-36	postive
	*CDK4*	0.632589072	9. 40E-43	postive
*RHNO1*	*ISG20L2*	0.626722824	9. 02E-42	postive
	*BIRC5*	0.632865496	8. 44E-43	postive
	*CDK4*	0.645286743	5. 93E-45	postive
*CDC25C*	*BIRC5*	0.736816364	1. 56E-64	postive
*PCNA*	*BIRC5*	0.659912223	1. 28E-47	postive
*CCNB1*	*HDAC1*	0.581785351	6. 80E-35	postive
	*BIRC5*	0.776756671	6. 83E-76	postive
	*CDK4*	0.581278611	8. 01E-35	postive
*E2F4*	*CDK4*	0.615042833	7. 07E-40	postive
*RFC2*	*BIRC5*	0.628600097	4. 40E-42	postive
*CHEK1*	*BIRC5*	0.706402415	3. 45E-57	postive
	*CDK4*	0.582160249	6. 02E-35	postive
*EXO1*	*BIRC5*	0.701060033	5. 39E-56	postive
*TOPBP1*	*PSMD2*	0.588023764	8. 74E-36	postive
	*ISG20L2*	0.61912506	1. 57E-40	postive
	*NRAS*	0.662623035	3. 94E-48	postive
	*CDK4*	0.586078393	1. 66E-35	postive
*MDM4*	*ISG20L2*	0.595493008	7. 06E-37	postive
*CSNK2A1*	*CDK4*	0.641629938	2. 61E-44	postive
*TAF4*	*ISG20L2*	0.630405859	2. 19E-42	postive
*RBBP4*	*HDAC1*	0.64857796	1. 53E-45	postive
	*NRAS*	0.647560256	2. 33E-45	postive
*RNF34*	*ISG20L2*	0.592032243	2. 28E-36	postive
	*HDAC1*	0.588319563	7. 92E-36	postive
	*NRAS*	0.605922057	1. 88E-38	postive
	*CDK4*	0.713701631	7. 29E-59	postive
*CDK1*	*BIRC5*	0.719246065	3. 59E-60	postive
*CASP2*	*ISG20L2*	0.644821505	7. 17E-45	postive
	*CDK4*	0.599367008	1. 87E-37	postive
*RFC5*	*BIRC5*	0.621470631	6. 56E-41	postive
	*CDK4*	0.587038164	1. 21E-35	postive
*SSRP1*	*BIRC5*	0.583951019	3. 35E-35	postive
	*CDK4*	0.676422514	8. 12E-51	postive
*FOXM1*	*BIRC5*	0.696564148	5. 20E-55	postive
	*CDK4*	0.609863356	4. 62E-39	postive
*TPX2*	*BIRC5*	0.76942673	1. 23E-73	postive
	*CDK4*	0.602969914	5. 32E-38	postive
*E2F8*	*BIRC5*	0.591834808	2. 44E-36	postive
*HDAC1*	*HDAC1*	0.956509886	5. 43E-199	postive
*KDM1A*	*HDAC1*	0.693016347	3. 02E-54	postive
*GTSE1*	*BIRC5*	0.686147713	8. 46E-53	postive
*CDK2*	*BIRC5*	0.595602449	6. 80E-37	postive
	*NRAS*	0.583681863	3. 66E-35	postive
	*CDK4*	0.656936615	4. 58E-47	postive
*RMI2*	*BIRC5*	0.618165641	2. 24E-40	postive
*AURKB*	*BIRC5*	0.615083631	6. 97E-40	postive
*ARID3A*	*NR6A1*	0.599385998	1. 85E-37	postive
*NDRG1*	*NDRG1*	0.7968711	1. 51E-82	postive

## Discussion

As a highly lethal malignant tumor, liver cancer has a large number of patients all over the world. Due to the high degree of heterogeneity of liver cancer, conventional indicators such as TMN staging, age and gender are slightly insufficient in predicting the prognosis of liver cancer patients. Anwanwan et al. reported that patients are often diagnosed with liver cancer in advanced stages, contributing to its poor prognosis (14). Of all liver cancer cases, 80% are hepatocellular carcinomas (HCCs) (2). Most patients with HCC are diagnosed by surveillance or incidental imaging analysis (15). Therefore, finding biomarkers with diagnostic significance and good predictive ability for the prognosis of liver cancer patients is an important research direction.

In addition, research in recent years found that the immune system plays a pivotal role in the maintenance of the integrity of an organism. Besides the protection against pathogens, it is strongly involved in cancer prevention, development and defense (16). The immune system has been shown to be a decisive factor in the occurrence and development of cancer (5). A variety of immune cells and immune-related molecules have been proved to be related to tumorigenesis, proliferation and development, for example, Yi L, Sun D, Han Q et al. found that interferon regulatory factor 3 can mediate the innate immune response and apoptosis of non-small cell lung cancer induced by Poly (16). Studying the role of immunity in tumors is of great significance, and immune-related genes are important research content. Some studies have found that immune genes are related to various biological behaviors such as tumor development, metastasis, and apoptosis (17, 18). At present, the gene signal based on abnormal mRNA has been used to predict the prognosis of hepatocellular carcinoma, which has great potential (19, 20). Gene signatures based on immune-related genes have been reported in a variety of cancers, such as lung squamous cell cancer, non-small cell lung cancer, esophageal carcinoma, etc (13, 21, 22). In this study, the high-throughput expression profiles of immune-related genes in TCGA-LIHC cohort were analyzed, and the differentially expressed immune genes were screened by differential analysis. After univariate cox regression analysis, lasso analysis and multivariate cox analysis, a five-gene signature (including *HDAC1*, *BIRC5*, *SPP1*, *STC2*, *NR6A1*) was constructed to predict the prognosis of hepatocellular carcinoma. The high expression of five genes was related to the poor prognosis of hepatocellular carcinoma patients. Calculate the patient’s risk values based on the relative expression levels of the five genes, and divide the patients into high-risk and low-risk groups based on the risk value. The results of survival analysis showed that the survival prognosis of patients in the high-risk group was significantly worse than that in the low-risk group. The AUC value of the ROC curve of the prognostic model was 0.764, indicating that the risk score prognostic model has a good predictive ability for survival prognosis. Univariate and multivariate independent prognostic analysis confirmed that the risk value based on this prognostic model can be used as an independent prognostic factor for hepatocellular carcinoma. In addition, in the independent hepatocellular carcinoma data set of the ICGC database, we conducted external verification of the prognosis model, and the predictive ability of the five-gene signature in the prognosis of hepatocellular carcinoma was further confirmed, and the expression levels of the five genes were verified.

Yamashita et al. reported that the immune cells within the tumor microenvironment (TME) play important roles in tumorigenesis. It has been known that these tumor associated immune cells may possess tumor-antagonizing or tumor-promoting functions (15). On the one hand, our study confirmed that the risk score based on the prognostic model has a significant correlation with the six immune cells through the TIMER database. On the other hand, the CIBERSORT algorithm was used to estimate the immune cell content of the TCGA-LIHC cohort samples and confirmed that the expression level of these five genes is related to the content of some immune cells.

Previous studies have shown that the five genes contained in the genetic signature can affect the occurrence and development of hepatocellular carcinoma. The full name of *HDAC1* is histone deacetylase 1, the protein encoded by this gene belongs to the histone deacetylase/acuc/apha family and is a component of the histone deacetylase complex. This complex is a key element in the control of cell proliferation and differentiation. *HDAC1* has been proved to be closely related to the occurrence and development of hepatocellular carcinoma. NoufAl-yhya et al. found that the application of *HDAC1* inhibitors can inhibit the proliferation and migration of hepatocellular carcinoma cells (23).

The human baculoviral IAP repeat containing 5 (*BIRC5*), also known as survivin, is a conserved member of the inhibitor of apoptosis protein (IAPs) family (24). It is reported that the increased expression of *BIRC5* in hepatocellular carcinoma inhibits the apoptosis of tumor cells, promotes the proliferation of tumor cells, and increases the resistance of hepatocellular carcinoma cells to radiotherapy and chemotherapy (25). TianQG et al. found that there was a positive correlation between the expression of *BIRC5* and *VEGF*, which could promote tumor angiogenesis (26). In addition, it is suggested that *BIRC5* can be used as a universal tumor antigen and a unique target for tumor immunotherapy (27).

Secreted phosphoprotein 1 (*SPP1*) also called as Osteopontin (*OPN*), it has been reported to be involved in tumor progression, metastasis and suggested as a promising prognosis/therapeutic target biomarker (28). Studies by AlexanderD.Nardo et al. have indicated that the imbalance of *SPP1* expression can promote the malignant development of hepatocellular carcinoma (29). LiguangYang et al. found that the overexpression of *SPP1* was associated with poor survival and could promote the proliferation of HCC cells (30).

Stanniocalcin 2 (*STC2*) is a glycoprotein hormone involved in many biological processes and a secretory protein that regulates malignant tumor progression. Studies by FanWu et al. have shown that down-regulation of *STC2* expression can inhibit the proliferation and survival of HCC cells (31). Recent studies have indicated that *STC2* plays an important role in the occurrence and development of hepatocellular carcinoma and contributes to the development of new HCC treatment strategies (32).

Although there are few related studies on Nuclear Receptor Subfamily 6 Group A Member 1 (*NR6A1*), the results of SunGD et al. confirmed that *NR6A1* plays an important role in the prognosis of patients with hepatocellular carcinoma (33).

In summary, the five genes that constitute the prognostic model of hepatocellular carcinoma may play an important role in the occurrence and development of tumors and have great research significance. The results of bioenrichment analysis of TCGA-LIHC cohort samples showed that 261 genes were enriched in the signal transduction pathway of P53 mediators. P53 is an important tumor suppressor gene, the P53-mediated cell signal transduction pathway plays an important role in regulating the normal life activities of cells, and its connection with other signal transduction pathways in the cell is very complicated. P53 mutation can play a protective role in inflammation and cancer (34). P53 plays an important regulatory role in blocking the cell cycle, promoting cell apoptosis, maintaining genome stability, and inhibiting tumor angiogenesis. The genes present in the signal transduction pathway of P53 mediators constitute a huge regulatory network, and some genes control the biological functions of a large number of related proteins (35). Muñoz-Fontela et al. have found that some genes in p53 pathway can participate in the process of immune regulation and play a role in the occurrence and development of tumors (36). In order to explore the potential relationship between these genes and prognostic-related immune genes, we conducted a correlation analysis of the two and constructed a regulatory network based on the results. From the network diagram, it can be seen that many genes in the signal transduction pathway of P53 mediators have a positive regulation relationship with some prognostic-related immune genes (*HDAC1 BIRC5*, *NR6A1*, *PSMD2*, *CDK4*, *ISG20L2*, *NDRG1*). Three genes in the prognostic model of hepatocellular carcinoma are involved in the construction of the regulatory network, which to a certain extent also shows that the five-gene signature has 3 great research value.

To our knowledge, the five-gene signature related prognostic model has not been reported, and may be able to provide effective strategies for the early diagnosis and treatment of hepatocellular carcinoma. Clinicians can calculate the risk score of each patient through the model, then select high-risk groups, and formulate treatment policies and strategies in advance according to the results. The calculation of risk score is based on the relative expression of genes, which is more practical in the process of diagnosis. Compared with conventional indicators, the prognostic model may be more accurate to predict the prognosis of patients.

However, it should be recognized that there are still some limitations in our study. First, our study is retrospective and needs to be further verified in prospective studies; Second, the model is mainly based on the data of the TCGA-LIHC cohort, the main races are white and black, and whether other races can be applied remains to be confirmed; Third, due to the lack of further functional experiments, the potential mechanism and interrelationship of the five genes need to be studied; finally, it is difficult to apply the risk score to clinical practice. In the follow-up research, we will further explore the predictive ability of the model, in-depth study of the potential mechanism of the five genes and the interaction between genes.

## Conclusion

In summary, our study established a new prognostic model of immune genes for hepatocellular carcinoma to predict the prognosis of patients, which may provide a potential target for clinical treatment of hepatocellular carcinoma. Our study also provides a new idea for the correlation between immune genes and the potential pathway of hepatocellular carcinoma.

## Data Availability Statement

‘The datasets presented in this study can be found in online repositories. The names of the repository/repositories and accession number(s) can be found in the article/**Supplementary Material**.

## Author Contributions

ZZ designed, analyzed the data, and write the manuscript. SC helped to prepare the dataset and participated in the discussion. WL and ML helped to search for some relevant papers for this research. MS analyzed the data and generated the figures and tables. BC guided the research process. All authors contributed to the article and approved the submitted version.

## Funding

This work was supported by the Quality Engineering Project of Anhui Province (No.:2020jyxm0898, No.:2020jyxm0910, No.:2019kfkc334), Clinical research project of Anhui Medical University(No.:2020xkj176), Soft health science research of Anhui province-Major project (No.:2020WR01003) and Entrepreneurship Project for College Students (No. 202010366032).

## Conflict of Interest

The authors declare that the research was conducted in the absence of any commercial or financial relationships that could be construed as a potential conflict of interest.

## Publisher’s Note

All claims expressed in this article are solely those of the authors and do not necessarily represent those of their affiliated organizations, or those of the publisher, the editors and the reviewers. Any product that may be evaluated in this article, or claim that may be made by its manufacturer, is not guaranteed or endorsed by the publisher.

## Supplementary Material

Supplementary Figure 1 Results of internal verification. **(A)** The KM- survival curve of TCGA-Train set and TCGA-Test set. **(B)** The ROC curve of TCGA-Train set and TCGA-Test set.

Supplementary Data Sheet 1 —The data of 11 genes obtained by lasso regression analysis.

Supplementary Data Sheet 2 —Risk value file for patients in the TCGA-LIHC cohort.

Supplementary Data Sheet 3 — Risk value file for patients in the ICGC liver cancer cohort.

Supplementary Data Sheet 4 —The data of 108 differentially expressed genes related to the signal transduction pathway of p53 mediators.

Supplementary Data Sheet 5 — Clinical information of patients in TCGA cohort.

Supplementary Data Sheet 6 — Clinical information of patients in ICGC cohort.

Supplementary Data Sheet 7 — Key codes applied in research.

## Abbreviations

TCGA, The Cancer Genome Atlas; ICGC, International Cancer Genome Consortium; HPA, Human Protein Atlas; TIMER, Tumor Immune Estimation Resource; FC, Fold Change; ROC, Receiver Operating Characteristic; GSEA, Gene Set Enrichment Analysis; PPI, Protein Protein Interaction; HCC, Hepatocellular Carcinoma; LIHC, Liver Hepatocellular Carcinoma; DEGs, Differentially Expressed Genes; OS, Overall Survival; FDR, False Discovery Rate; NES, Normalized Enrichment Score; AFP, Alpha-fetoprotein; NLR, Neutrophil-to-Lymphocyte Ratio; GPC3, Glypican-3.
