# Supplementary figures and images for: Identification, Verification and Pathway Enrichment Analysis of Prognosis-Related Immune Genes in Patients With Hepatocellular Carcinoma
Source: Front Oncol. 2021 Sep 20;11:695001. doi: 10.3389/fonc.2021.695001 (PMC8488301; doi:10.3389/fonc.2021.695001)

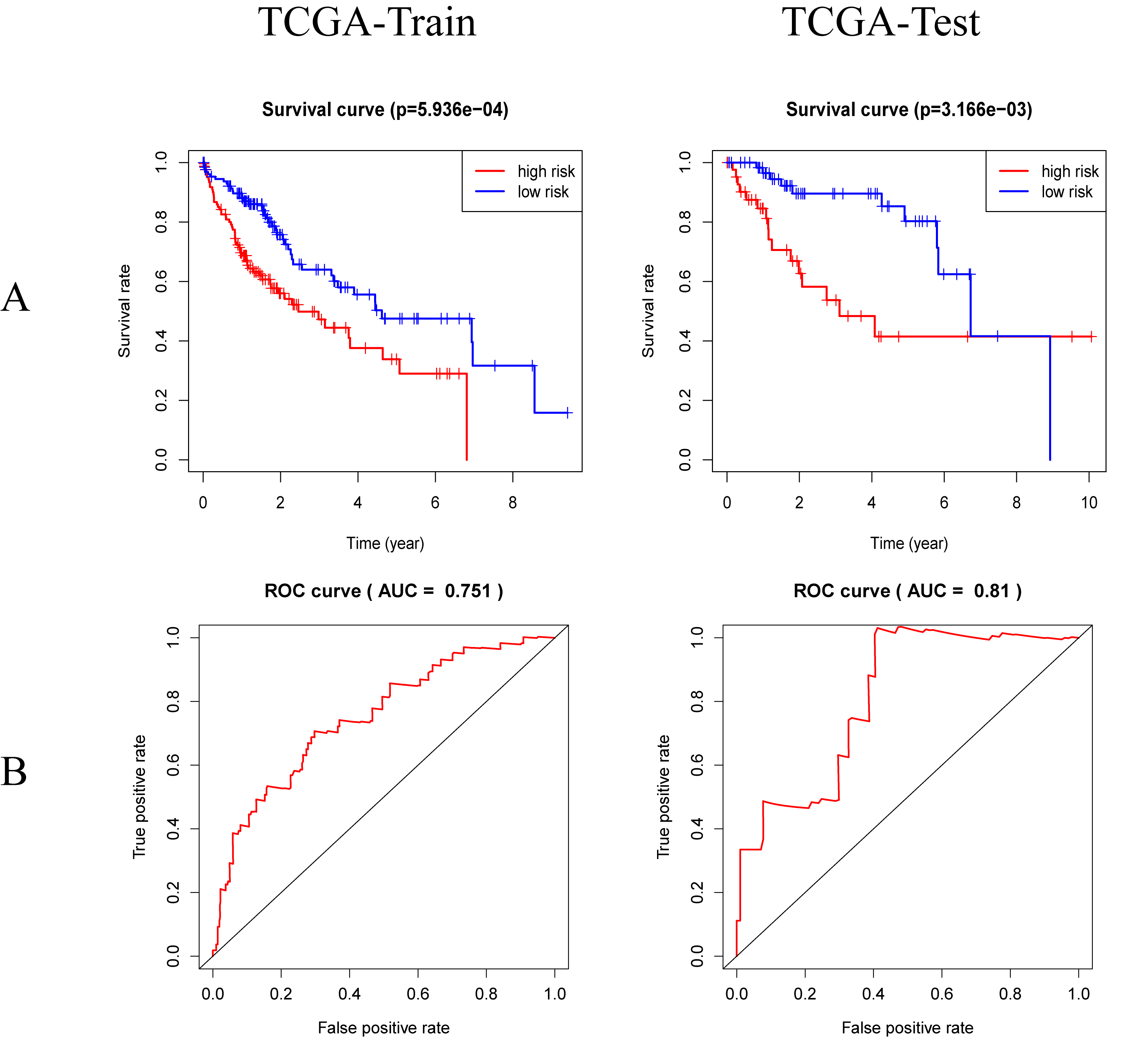

Supplement: Supplementary Figure 1 — Results of internal verification. (A) The KM- survival curve of TCGA-Train set and TCGA-Test set. (B) The ROC curve of TCGA-Train set and TCGA-Test set. [file Image_1.tif]
