# Supplementary material for: Identification, Verification and Pathway Enrichment Analysis of Prognosis-Related Immune Genes in Patients With Hepatocellular Carcinoma
Source: Front Oncol. 2021 Sep 20;11:695001. doi: 10.3389/fonc.2021.695001 (PMC8488301; doi:10.3389/fonc.2021.695001)
Supplement: Supplementary Data Sheet 7 — — Key codes applied in research. [file DataSheet_7.docx]

**Differential analysis of gene expression:**

setwd("E:\\TCGA ICGC\\liver\\09.immuneDiff")

fdrFilter=0.05 logFCfilter=1 conNum=50 treatNum=374

rt=read.table("all.txt",sep="\t",header=T,check.names=F,row.names=1)

diffExp=read.table("diffGeneExp.txt",sep="\t",header=T,check.names=F,row.names=1)

gene=read.table("gene.txt",sep="\t",header=F)

immuneDiffAll=rt[intersect(gene[,1],row.names(rt)),]

immuneDiffGene=intersect(gene[,1],row.names(diffExp))

hmExp=diffExp[immuneDiffGene,]

immuneDiffResult=immuneDiffAll[immuneDiffGene,]

immuneDiffResult=rbind(ID=colnames(immuneDiffResult),immuneDiffResult)

write.table(immuneDiffResult,file="immuneDiff.xls",sep="\t",col.names=F,quote=F)

immuneGeneExp=rbind(ID=colnames(hmExp),hmExp)

write.table(immuneGeneExp,file="immuneGeneExp.txt",sep="\t",col.names=F,quote=F)

pdf(file="vol.pdf",height=5,width=5)

xMax=max(abs(as.numeric(as.vector(immuneDiffAll$logFC))))

yMax=max(-log10(immuneDiffAll$fdr))+1

plot(as.numeric(as.vector(immuneDiffAll$logFC)), -log10(immuneDiffAll$fdr), xlab="logFC",ylab="-log10(fdr)",

main="Volcano", ylim=c(0,yMax),xlim=c(-xMax,xMax),yaxs="i",pch=20, cex=0.8)

diffSub=subset(immuneDiffAll, fdr<fdrFilter & as.numeric(as.vector(logFC))>logFCfilter)

points(as.numeric(as.vector(diffSub$logFC)), -log10(diffSub$fdr), pch=20, col="red",cex=0.8)

diffSub=subset(immuneDiffAll, fdr<fdrFilter & as.numeric(as.vector(logFC))<(-logFCfilter))

points(as.numeric(as.vector(diffSub$logFC)), -log10(diffSub$fdr), pch=20, col="green",cex=0.8)

abline(v=0,lty=2,lwd=3)

dev.off()

library(pheatmap)

hmExp=log2(hmExp+0.001)

Type=c(rep("N",conNum),rep("T",treatNum))

names(Type)=colnames(diffExp)

Type=as.data.frame(Type)

pdf(file="heatmap.pdf",height=12,width=15)

pheatmap(hmExp,

annotation=Type,

color = colorRampPalette(c("green", "black", "red"))(50),

cluster_cols =F,

show_colnames = F,

show_rownames = F,

fontsize = 12,

fontsize_row=3,

fontsize_col=10)

dev.off()

**Univariate regression analysis:**

library(survival)

pFilter=0.0001

setwd("E:\\TCGA ICGC\\liver\\16.uniCox")

rt=read.table("expTime.txt",header=T,sep="\t",check.names=F,row.names=1)

outTab=data.frame()

sigGenes=c("futime","fustat")

for(i in colnames(rt[,3:ncol(rt)])){

cox <- coxph(Surv(futime, fustat) ~ rt[,i], data = rt)

coxSummary = summary(cox)

coxP=coxSummary$coefficients[,"Pr(>|z|)"]

if(coxP<pFilter){

sigGenes=c(sigGenes,i)

outTab=rbind(outTab,

cbind(id=i,

HR=coxSummary$conf.int[,"exp(coef)"],

HR.95L=coxSummary$conf.int[,"lower .95"],

HR.95H=coxSummary$conf.int[,"upper .95"],

pvalue=coxSummary$coefficients[,"Pr(>|z|)"])

)

}

}

write.table(outTab,file="uniCox.txt",sep="\t",row.names=F,quote=F)

uniSigExp=rt[,sigGenes]

uniSigExp=cbind(id=row.names(uniSigExp),uniSigExp)

write.table(uniSigExp,file="uniSigExp.txt",sep="\t",row.names=F,quote=F)

rt <- read.table("uniCox.txt",header=T,sep="\t",row.names=1,check.names=F)

gene <- rownames(rt)

hr <- sprintf("%.3f",rt$"HR")

hrLow <- sprintf("%.3f",rt$"HR.95L")

hrHigh <- sprintf("%.3f",rt$"HR.95H")

Hazard.ratio <- paste0(hr,"(",hrLow,"-",hrHigh,")")

pVal <- ifelse(rt$pvalue<0.0001, "<0.0001", sprintf("%.3f", rt$pvalue))

pdf(file="forest.pdf", width = 8,height = 6.5)

n <- nrow(rt)

nRow <- n+1

ylim <- c(1,nRow)

layout(matrix(c(1,2),nc=2),width=c(3,2.5))

xlim = c(0,3)

par(mar=c(4,2.5,2,1))

plot(1,xlim=xlim,ylim=ylim,type="n",axes=F,xlab="",ylab="")

text.cex=0.8

text(0,n:1,gene,adj=0,cex=text.cex)

text(1.5-0.5*0.2,n:1,pVal,adj=1,cex=text.cex);text(1.5-0.5*0.2,n+1,'pvalue',cex=text.cex,font=2,adj=1)

text(3,n:1,Hazard.ratio,adj=1,cex=text.cex);text(3,n+1,'Hazard ratio',cex=text.cex,font=2,adj=1,)

par(mar=c(4,1,2,1),mgp=c(2,0.5,0))

xlim = c(0,max(as.numeric(hrLow),as.numeric(hrHigh)))

plot(1,xlim=xlim,ylim=ylim,type="n",axes=F,ylab="",xaxs="i",xlab="Hazard ratio")

arrows(as.numeric(hrLow),n:1,as.numeric(hrHigh),n:1,angle=90,code=3,length=0.05,col="darkblue",lwd=2.5)

abline(v=1,col="black",lty=2,lwd=2)

boxcolor = ifelse(as.numeric(hr) > 1, 'red', 'green')

points(as.numeric(hr), n:1, pch = 15, col = boxcolor, cex=1.3)

axis(1)

dev.off()

**Lasso regression analysis:**

library("glmnet")

library("survival")

setwd("E:\\TCGA ICGC\\liver\\16.lasso") rt=read.table("uniSigExp.txt",header=T,sep="\t",row.names=1,check.names=F) rt$futime[rt$futime<=0]=1

x=as.matrix(rt[,c(3:ncol(rt))])

y=data.matrix(Surv(rt$futime,rt$fustat))

fit <- glmnet(x, y, family = "cox", maxit = 1000)

pdf("lambda.pdf")

plot(fit, xvar = "lambda", label = TRUE)

dev.off()

cvfit <- cv.glmnet(x, y, family="cox", maxit = 1000)

pdf("cvfit.pdf")

plot(cvfit)

abline(v=log(c(cvfit$lambda.min,cvfit$lambda.1se)),lty="dashed")

dev.off()

coef <- coef(fit, s = cvfit$lambda.min)

index <- which(coef != 0)

actCoef <- coef[index]

lassoGene=row.names(coef)[index]

lassoGene=c("futime","fustat",lassoGene)

lassoSigExp=rt[,lassoGene]

lassoSigExp=cbind(id=row.names(lassoSigExp),lassoSigExp)

write.table(lassoSigExp,file="lassoSigExp.txt",sep="\t",row.names=F,quote=F)

**Multivariate regression analysis:**

library(survival)

library(survminer)

setwd("E:\\TCGA ICGC\\liver\\20.multiCox") rt=read.table("train.txt",header=T,sep="\t",check.names=F,row.names=1) rt[,"futime"]=rt[,"futime"]/365

multiCox=coxph(Surv(futime, fustat) ~ ., data = rt)

multiCox=step(multiCox,direction = "both")

multiCoxSum=summary(multiCox)

outTab=data.frame()

outTab=cbind(

coef=multiCoxSum$coefficients[,"coef"],

HR=multiCoxSum$conf.int[,"exp(coef)"],

HR.95L=multiCoxSum$conf.int[,"lower .95"],

HR.95H=multiCoxSum$conf.int[,"upper .95"],

pvalue=multiCoxSum$coefficients[,"Pr(>|z|)"])

outTab=cbind(id=row.names(outTab),outTab)

write.table(outTab,file="multiCox.xls",sep="\t",row.names=F,quote=F)

pdf(file="forest.pdf",

width = 8,

height = 5,

)

ggforest(multiCox,

main = "Hazard ratio",

cpositions = c(0.02,0.22, 0.4),

fontsize = 0.7,

refLabel = "reference",

noDigits = 2)

dev.off()

riskScore=predict(multiCox,type="risk",newdata=rt)

coxGene=rownames(multiCoxSum$coefficients)

coxGene=gsub("`","",coxGene)

outCol=c("futime","fustat",coxGene)

medianTrainRisk=median(riskScore)

risk=as.vector(ifelse(riskScore>medianTrainRisk,"high","low"))

write.table(cbind(id=rownames(cbind(rt[,outCol],riskScore,risk)),cbind(rt[,outCol],riskScore,risk)),

file="riskTrain.txt",

sep="\t",

quote=F,

row.names=F)

rtTest=read.table("test.txt",header=T,sep="\t",check.names=F,row.names=1) rtTest[,"futime"]=rtTest[,"futime"]/365

riskScoreTest=predict(multiCox,type="risk",newdata=rtTest) riskTest=as.vector(ifelse(riskScoreTest>medianTrainRisk,"high","low"))

write.table(cbind(id=rownames(cbind(rtTest[,outCol],riskScoreTest,riskTest)),cbind(rtTest[,outCol],riskScore=riskScoreTest,risk=riskTest)),

file="riskTest.txt",

sep="\t",

quote=F,

row.names=F)

**Construct the survival curve:**

setwd("E:\\TCGA ICGC\\liver\\21.survival")

library(survival)

library("survminer")

rt=read.table("riskTest.txt",header=T,sep="\t")

diff=survdiff(Surv(futime, fustat) ~risk,data = rt)

pValue=1-pchisq(diff$chisq,df=1)

pValue=signif(pValue,4)

pValue=format(pValue, scientific = TRUE)

fit <- survfit(Surv(futime, fustat) ~ risk, data = rt)

pdf(file="survival.pdf",onefile = FALSE,

width = 5.5,

height =5)

ggsurvplot(fit,

data=rt,

conf.int=TRUE,

pval=paste0("p=",pValue),

pval.size=4,

risk.table=TRUE,

legend.labs=c("High risk", "Low risk"),

legend.title="Risk",

xlab="Time(years)",

break.time.by = 1,

risk.table.title="",

palette=c("red", "blue"),

risk.table.height=.25)

dev.off()

summary(fit)

**Construct ROC curve:**

library(survivalROC)

setwd("E:\\TCGA ICGC\\liver\\22.ROC")

rt=read.table("riskTrain.txt",header=T,sep="\t",check.names=F,row.names=1)

pdf(file="rocTrain.pdf",width=6,height=6)

par(oma=c(0.5,1,0,1),font.lab=1.5,font.axis=1.5)

roc=survivalROC(Stime=rt$futime, status=rt$fustat, marker = rt$riskScore,

predict.time =1, method="KM")

plot(roc$FP, roc$TP, type="l", xlim=c(0,1), ylim=c(0,1),col='red',

xlab="False positive rate", ylab="True positive rate",

main=paste("ROC curve (", "AUC = ",round(roc$AUC,3),")"),

lwd = 2, cex.main=1.3, cex.lab=1.2, cex.axis=1.2, font=1.2)

abline(0,1)

dev.off()

rt=read.table("riskTest.txt",header=T,sep="\t",check.names=F,row.names=1)

pdf(file="rocTest.pdf",width=6,height=6)

par(oma=c(0.5,1,0,1),font.lab=1.5,font.axis=1.5)

roc=survivalROC(Stime=rt$futime, status=rt$fustat, marker = rt$riskScore,

predict.time =1, method="KM")

plot(roc$FP, roc$TP, type="l", xlim=c(0,1), ylim=c(0,1),col='red',

xlab="False positive rate", ylab="True positive rate",

main=paste("ROC curve (", "AUC = ",round(roc$AUC,3),")"),

lwd = 2, cex.main=1.3, cex.lab=1.2, cex.axis=1.2, font=1.2)

abline(0,1)

dev.off()

**Independent prognostic analysis:**

library(survival)

setwd("E:\\TCGA ICGC\\liver\\32.TCGAcox\\26.uniIndep") rt=read.table("indepInput.txt",header=T,sep="\t",check.names=F,row.names=1)

outTab=data.frame()

for(i in colnames(rt[,3:ncol(rt)])){

cox <- coxph(Surv(futime, fustat) ~ rt[,i], data = rt)

coxSummary = summary(cox)

coxP=coxSummary$coefficients[,"Pr(>|z|)"]

outTab=rbind(outTab,

cbind(id=i,

HR=coxSummary$conf.int[,"exp(coef)"],

HR.95L=coxSummary$conf.int[,"lower .95"],

HR.95H=coxSummary$conf.int[,"upper .95"],

pvalue=coxSummary$coefficients[,"Pr(>|z|)"])

)

}

write.table(outTab,file="uniCox.txt",sep="\t",row.names=F,quote=F)

rt <- read.table("uniCox.txt",header=T,sep="\t",row.names=1,check.names=F)

gene <- rownames(rt)

hr <- sprintf("%.3f",rt$"HR")

hrLow <- sprintf("%.3f",rt$"HR.95L")

hrHigh <- sprintf("%.3f",rt$"HR.95H")

Hazard.ratio <- paste0(hr,"(",hrLow,"-",hrHigh,")")

pVal <- ifelse(rt$pvalue<0.001, "<0.001", sprintf("%.3f", rt$pvalue))

pdf(file="forest1.pdf", width = 7,height = 4)

n <- nrow(rt)

nRow <- n+1

ylim <- c(1,nRow)

layout(matrix(c(1,2),nc=2),width=c(3,2.5))

xlim = c(0,3)

par(mar=c(4,2.5,2,1))

plot(1,xlim=xlim,ylim=ylim,type="n",axes=F,xlab="",ylab="")

text.cex=0.8

text(0,n:1,gene,adj=0,cex=text.cex)

text(1.5-0.5*0.2,n:1,pVal,adj=1,cex=text.cex);text(1.5-0.5*0.2,n+1,'pvalue',cex=text.cex,font=2,adj=1)

text(3,n:1,Hazard.ratio,adj=1,cex=text.cex);text(3,n+1,'Hazard ratio',cex=text.cex,font=2,adj=1,)

par(mar=c(4,1,2,1),mgp=c(2,0.5,0))

xlim = c(0,max(as.numeric(hrLow),as.numeric(hrHigh)))

plot(1,xlim=xlim,ylim=ylim,type="n",axes=F,ylab="",xaxs="i",xlab="Hazard ratio")

arrows(as.numeric(hrLow),n:1,as.numeric(hrHigh),n:1,angle=90,code=3,length=0.05,col="darkblue",lwd=2.5)

abline(v=1,col="black",lty=2,lwd=2)

boxcolor = ifelse(as.numeric(hr) > 1, 'red', 'green')

points(as.numeric(hr), n:1, pch = 15, col = boxcolor, cex=1.3)

axis(1)

dev.off()

library(survival)

setwd("E:\\TCGA ICGC\\liver\\32.TCGAcox\\27.multiIndep")

rt=read.table("indepInput.txt",header=T,sep="\t",check.names=F,row.names=1)

multiCox=coxph(Surv(futime, fustat) ~ ., data = rt)

multiCoxSum=summary(multiCox)

outTab=data.frame()

outTab=cbind(

HR=multiCoxSum$conf.int[,"exp(coef)"],

HR.95L=multiCoxSum$conf.int[,"lower .95"],

HR.95H=multiCoxSum$conf.int[,"upper .95"],

pvalue=multiCoxSum$coefficients[,"Pr(>|z|)"])

outTab=cbind(id=row.names(outTab),outTab)

write.table(outTab,file="multiCox.xls",sep="\t",row.names=F,quote=F)

rt <- read.table("multiCox.xls",header=T,sep="\t",row.names=1,check.names=F)

gene <- rownames(rt)

hr <- sprintf("%.3f",rt$"HR")

hrLow <- sprintf("%.3f",rt$"HR.95L")

hrHigh <- sprintf("%.3f",rt$"HR.95H")

Hazard.ratio <- paste0(hr,"(",hrLow,"-",hrHigh,")")

pVal <- ifelse(rt$pvalue<0.001, "<0.001", sprintf("%.3f", rt$pvalue))

pdf(file="forest.pdf", width = 7,height = 4)

n <- nrow(rt)

nRow <- n+1

ylim <- c(1,nRow)

layout(matrix(c(1,2),nc=2),width=c(3,2.5))

xlim = c(0,3)

par(mar=c(4,2.5,2,1))

plot(1,xlim=xlim,ylim=ylim,type="n",axes=F,xlab="",ylab="")

text.cex=0.8

text(0,n:1,gene,adj=0,cex=text.cex)

text(1.5-0.5*0.2,n:1,pVal,adj=1,cex=text.cex);text(1.5-0.5*0.2,n+1,'pvalue',cex=text.cex,font=2,adj=1)

text(3,n:1,Hazard.ratio,adj=1,cex=text.cex);text(3,n+1,'Hazard ratio',cex=text.cex,font=2,adj=1,)

par(mar=c(4,1,2,1),mgp=c(2,0.5,0))

xlim = c(0,max(as.numeric(hrLow),as.numeric(hrHigh)))

plot(1,xlim=xlim,ylim=ylim,type="n",axes=F,ylab="",xaxs="i",xlab="Hazard ratio")

arrows(as.numeric(hrLow),n:1,as.numeric(hrHigh),n:1,angle=90,code=3,length=0.05,col="darkblue",lwd=2.5)

abline(v=1,col="black",lty=2,lwd=2)

boxcolor = ifelse(as.numeric(hr) > 1, 'red', 'green')

points(as.numeric(hr), n:1, pch = 15, col = boxcolor, cex=1.3)

axis(1)

dev.off()

**Immune infiltration analysis:**

library("limma")

expFile="symbol.txt"

setwd("E:\\TCGA ICGC\\liver\\27.免疫浸润\\30")

rt=read.table(expFile,sep="\t",header=T,check.names=F)

rt=as.matrix(rt)

rownames(rt)=rt[,1]

exp=rt[,2:ncol(rt)]

dimnames=list(rownames(exp),colnames(exp))

data=matrix(as.numeric(as.matrix(exp)),nrow=nrow(exp),dimnames=dimnames)

data=avereps(data)

group=sapply(strsplit(colnames(data),"\\-"),"[",4)

group=sapply(strsplit(group,""),"[",1)

group=gsub("2","1",group)

data=data[,group==0]

data=data[rowMeans(data)>0,]

v <-voom(data, plot = F, save.plot = F)

out=v$E

out=rbind(ID=colnames(out),out)

write.table(out,file="uniq.symbol.txt",sep="\t",quote=F,col.names=F)

source("TMEimmune31.CIBERSORT.R")

results=CIBERSORT("ref.txt", "uniq.symbol.txt", perm=100, QN=TRUE)

library(corrplot)

input="CIBERSORT-Results.txt"

outpdf="barplot.pdf"

pFilter=0.05

setwd("E:\\TCGA ICGC\\liver \\31")

immune=read.table("CIBERSORT-Results.txt",sep="\t",header=T,row.names=1,check.names=F)

immune=immune[immune[,"P-value"]<pFilter,]

immune=as.matrix(immune[,1:(ncol(immune)-3)])

data=t(immune)

col=rainbow(nrow(data),s=0.7,v=0.7)

pdf(outpdf,height=10,width=22)

par(las=1,mar=c(8,5,4,16),mgp=c(3,0.1,0),cex.axis=1.5)

a1 = barplot(data,col=col,yaxt="n",ylab="Relative Percent",xaxt="n",cex.lab=1.8)

a2=axis(2,tick=F,labels=F)

axis(2,a2,paste0(a2*100,"%"))

axis(1,a1,labels=F)

par(srt=60,xpd=T);text(a1,-0.02,colnames(data),adj=1,cex=0.6);par(srt=0)

ytick2 = cumsum(data[,ncol(data)])

ytick1 = c(0,ytick2[-length(ytick2)])

legend(par('usr')[2]*0.98,par('usr')[4],legend=rownames(data),col=col,pch=15,bty="n",cex=1.3)

dev.off()

pdf("corrplot.pdf",height=14,width=14)

par(oma=c(0.5,1,1,1.2))

M=cor(immune)

corrplot(M, order = "AOE", type = "upper", tl.pos = "lt")

corrplot(M, add = TRUE, type = "lower", method = "number", order = "AOE",

col = "black", diag = FALSE, tl.pos = "n", cl.pos = "n")

dev.off()

library(limma)

library(vioplot)

immuneFile="CIBERSORT-Results.txt"

expFile="symbol.txt"

gene="CTCR4"

pFilter=0.05

setwd("E:\\TCGA ICGC\\liver\\32")

immune=read.table(immuneFile,sep="\t",header=T,row.names=1,check.names=F)

immune=immune[immune[,"P-value"]<pFilter,]

immune=as.matrix(immune[,1:(ncol(immune)-3)])

rt=read.table(expFile,sep="\t",header=T,check.names=F)

rt=as.matrix(rt)

rownames(rt)=rt[,1]

exp=rt[,2:ncol(rt)]

dimnames=list(rownames(exp),colnames(exp))

data=matrix(as.numeric(as.matrix(exp)),nrow=nrow(exp),dimnames=dimnames)

data=avereps(data)

group=sapply(strsplit(colnames(data),"\\-"),"[",4)

group=sapply(strsplit(group,""),"[",1)

group=gsub("2","1",group)

data=data[,group==0]

lowName=colnames(data)[data[gene,]<=median(data[gene,])]

highName=colnames(data)[data[gene,]>median(data[gene,])]

lowImm=intersect(row.names(immune),lowName)

highImm=intersect(row.names(immune),highName)

rt=rbind(immune[lowImm,],immune[highImm,])

lowNum=length(lowImm)

highNum=length(highImm)

outTab=data.frame()

pdf("vioplot.pdf",height=8,width=13)

par(las=1,mar=c(10,6,3,3))

x=c(1:ncol(rt))

y=c(1:ncol(rt))

plot(x,y,

xlim=c(0,63),ylim=c(min(rt),max(rt)+0.02),

main="",xlab="", ylab="Fraction",

pch=21,

col="white",

xaxt="n")

for(i in 1:ncol(rt)){

if(sd(rt[1:lowNum,i])==0){

rt[1,i]=0.001

}

if(sd(rt[(lowNum+1):(lowNum+highNum),i])==0){

rt[(lowNum+1),i]=0.001

}

lowData=rt[1:lowNum,i]

highData=rt[(lowNum+1):(lowNum+highNum),i]

vioplot(lowData,at=3*(i-1),lty=1,add = T,col = 'green')

vioplot(highData,at=3*(i-1)+1,lty=1,add = T,col = 'red')

wilcoxTest=wilcox.test(lowData,highData)

p=wilcoxTest$p.value

if(p<pFilter){

cellPvalue=cbind(Cell=colnames(rt)[i],pvalue=p)

outTab=rbind(outTab,cellPvalue)

}

mx=max(c(lowData,highData))

lines(c(x=3*(i-1)+0.2,x=3*(i-1)+0.8),c(mx,mx))

text(x=3*(i-1)+0.5, y=mx+0.02, labels=ifelse(p<0.001, paste0("p<0.001"), paste0("p=",sprintf("%.03f",p))), cex = 0.8)

}

text(seq(1,64,3),-0.05,xpd = NA,labels=colnames(rt),cex = 1,srt = 45,pos=2)

dev.off()

write.table(outTab,file="diff.result.txt",sep="\t",row.names=F,quote=F)

setwd("E:\\TCGA ICGC\\liver \\immuneCor ")

TIMER = read.table("immuneEstimation.txt", row.names=1 ,header=T,sep="\t",check.names=F)

immuneScore = read.table("riskTrain.txt", row.names=1 ,header=T,sep="\t",check.names=F) immuneScore=t(immuneScore[,3:(ncol(immuneScore)-1)])

TIMER=t(TIMER)

group=sapply(strsplit(colnames(TIMER),"\\-"),"[",4)

group=sapply(strsplit(group,""),"[",1)

group=gsub("2","1",group)

TIMER=TIMER[,group==0]

colnames(TIMER)=gsub("(.*?)\\-(.*?)\\-(.*?)\\-.*","\\1\\-\\2\\-\\3",colnames(TIMER))

sameSample=intersect(colnames(TIMER),colnames(immuneScore))

TIMER1=TIMER[,sameSample]

immuneScore1=immuneScore[,sameSample]

outTab=data.frame()

i="riskScore"

for(j in row.names(TIMER1)){

x=as.numeric(immuneScore1[i,])

y=as.numeric(TIMER1[j,])

corT=cor.test(x,y)

cor=sprintf("%.03f",corT$estimate)

pvalue=corT$p.value

z=lm(y~x)

pval=0

if(pvalue<0.001){

pval=signif(pvalue,4)

pval=format(pval, scientific = TRUE)

}else{

pval=sprintf("%.03f",pvalue)

}

pdf(file=paste0(j,".pdf"),height=4.5,width=4.5)

plot(x,y, type="p",pch=16,col="blue",main=paste("Cor=",cor," (p=",pval,")",sep=""),

cex=0.8, cex.lab=1.2, cex.main=1.2,cex.axis=1, xlab="Risk score",xlim=c(0,10),

ylab=j)

lines(x,fitted(z),col=2)

dev.off()

}

**Internal verification:**

setwd("E:\\TCGA ICGC\\liver\\32.TCGAcox\\27.multiIndep ")

rt=read.table("expTime.txt",sep="\t",header=T,check.names=F)

library(caret)

inTrain<-createDataPartition(y=rt[,3],p=0.7,list=F)

train<-rt[inTrain,]

test<-rt[-inTrain,]

write.table(train,file="train.txt",sep="\t",quote=F,row.names=F)

write.table(test,file="test.txt",sep="\t",quote=F,row.names=F)

setwd("E:\\TCGA ICGC\\liver\\21.survival")

library(survival)

library("survminer")

rt=read.table("riskTest.txt",header=T,sep="\t")

diff=survdiff(Surv(futime, fustat) ~risk,data = rt)

pValue=1-pchisq(diff$chisq,df=1)

pValue=signif(pValue,4)

pValue=format(pValue, scientific = TRUE)

fit <- survfit(Surv(futime, fustat) ~ risk, data = rt)

pdf(file="survival.pdf",onefile = FALSE,

width = 5.5,

height =5)

ggsurvplot(fit,

data=rt,

conf.int=TRUE,

pval=paste0("p=",pValue),

pval.size=4,

risk.table=TRUE,

legend.labs=c("High risk", "Low risk"),

legend.title="Risk",

xlab="Time(years)",

break.time.by = 1,

risk.table.title="",

palette=c("red", "blue"),

risk.table.height=.25)

dev.off()

summary(fit)

library(survivalROC)

setwd("E:\\TCGA ICGC\\liver\\22.ROC")

rt=read.table("riskTrain.txt",header=T,sep="\t",check.names=F,row.names=1)

pdf(file="rocTrain.pdf",width=6,height=6)

par(oma=c(0.5,1,0,1),font.lab=1.5,font.axis=1.5)

roc=survivalROC(Stime=rt$futime, status=rt$fustat, marker = rt$riskScore,

predict.time =1, method="KM")

plot(roc$FP, roc$TP, type="l", xlim=c(0,1), ylim=c(0,1),col='red',

xlab="False positive rate", ylab="True positive rate",

main=paste("ROC curve (", "AUC = ",round(roc$AUC,3),")"),

lwd = 2, cex.main=1.3, cex.lab=1.2, cex.axis=1.2, font=1.2)

abline(0,1)

dev.off()

rt=read.table("riskTest.txt",header=T,sep="\t",check.names=F,row.names=1)

pdf(file="rocTest.pdf",width=6,height=6)

par(oma=c(0.5,1,0,1),font.lab=1.5,font.axis=1.5)

roc=survivalROC(Stime=rt$futime, status=rt$fustat, marker = rt$riskScore,

predict.time =1, method="KM")

plot(roc$FP, roc$TP, type="l", xlim=c(0,1), ylim=c(0,1),col='red',

xlab="False positive rate", ylab="True positive rate",

main=paste("ROC curve (", "AUC = ",round(roc$AUC,3),")"),

lwd = 2, cex.main=1.3, cex.lab=1.2, cex.axis=1.2, font=1.2)

abline(0,1)

dev.off()
